# A Flow Cytometry Panel for Differential Diagnosis of Mantle Cell Lymphoma from Atypical B-Chronic Lymphocytic Leukaemia

**DOI:** 10.52547/ibj.3275

**Published:** 2022-02-16

**Authors:** Mahdieh Mehrpouri, Maryam Sadat Hosseini, Leila Jafari, Mohammad Mosleh, Eesmaeil Shahabi Satlsar

**Affiliations:** 1 Department of Laboratory Sciences, School of Allied Medical Sciences, Alborz University of Medical Sciences, Karaj, Iran;; 2 Department of Hematology and Blood Banking, School of Allied Medical Sciences, Shahid Beheshti University of Medical Sciences, Tehran, Iran;; 3 School of paramedical Sciences, Guilan University of Medical Sciences, Rasht, Iran

**Keywords:** Chronic lymphocytic leukemia, Flow cytometry, Immunoglobulin light chain, Lymphoma

## Abstract

**Background:**

Differential diagnosis of CLDs has remained challenging due to the highly variable morphology features and immunophenotyping. Currently, the development of multiple-marker panel analyses by flow cytometry has opened a broad way for diagnosis of CLDs.

**Methods:**

We analyzed the peripheral blood and BM samples of 131 patients with B-cell CLDs (including 91 CLL, 15 atypical CLL, 14 MCL, and 11 CD5-/CD10-lymphoma patients) from April 2018 to April 2019, using a panel of specific markers by flow cytometry.

**Results:**

Our results indicated that the expression pattern of CD22, CD23, FMC-7, and CD5 allowed us to accurately and differentially diagnose the B-CLL, MCL, and CD5-/CD10- lymphoma, while it was not capable of differentiating MCL from atypical CLL. We, however, found that the expression patterns of CD38 and immunoglobulin light chain differed significantly between atypical B-CLL and MCL. CD38 and lambda light chain were remarkably expressed in MCL patients (92.8% and 85%, respectively) compared to the atypical CLL (1.1% and 0% respectively), with the *p* value less than 0.001 for both markers. In contrast to MCL patients, all the patients with atypical CLL, expressed kappa light chain. The IHC method used for cyclin D1 confirmed that the flow cytometry detection of kappa and lambda light chains could provide a new approach with high sensitivity (91%) and moderate specificity (50%) to distinguish MCL patients from atypical B-CLL.

**Conclusion:**

Expression of CD5, CD20 (bright), CD22, FMC-7, CD38, and lambda light chain with no expression of CD23 can accurately detect MCL and differentiate it from atypical B-CLL.

## INTRODUCTION

Chronic lymphoproliferative disorders are a group of heterogeneous diseases that require more specific targeted and therapeutic strategies. Novel technical advances have recently enabled clinicians to accurately diagnose such disorders. While clinical characteristics, morphologic features, and immunologic phenotypes provide reliable diagnosis in most cases of B-cell CLDs, a small subset of these malignancies shows a variable phenotype that makes the diagnosis challenging. Thus, the immunophenotypic characterization of cancer cells using MoAbs targeting specific CD markers, which are basically applied to characterize the chronic leukemia, has earned a crucial role in differential diagnosis of CLDs^[^^1^^-^^3^^]^. The flow cytometric analysis is nowadays considered as an accurate and a precise diagnostic tool for this purpose^[^^4^^-^^7^^]^. However, since no marker offers an absolute diagnostic value, applying a panel of MoAbs to create scoring systems are assumed to provide more beneficial diagnostic insight rather than single antigens^[^^8^^,^^9^^]^.

In case of CLL, heterogeneous morphologic and immunologic characteristics have long been described. Moreover, its clinical features are highly variable, which are even observed within the same clinical stage. Based on the French-American-British classification and morphologic attributes, B-CLL is subdivided into typical composed of monomorphic lymphocytes and atypical (or B-CLL variant) with mixed cell types^[^^2^^]^. Although small lymphocytes with easily diagnosable clumped chromatin and specific immunophenotype make the diagnosis of classical B-CLL quite clear, the atypical B-CLL with its highly variable morphology and immunophenotype is faced with more challenges to be precisely diagnosed. 

MCL is characterized by the proliferation of B cells in the mantle zone of lymphoid follicles^[^^10^^]^. Morphologically, MCL patients show a monotonous proliferation of lymphocytes, which contain scant cytoplasm and slightly irregular nuclei. Immunophenotype of MCL cells is also characterized by the co-expression of CD5 and pan B-cell antigens (CD19, CD20, CD22, and CD24), which are also detectable in CLL/small lymphocytic lymphoma. While some believe that CD23 might offer a distinguishing value, several contradictory results have been reported in this case^[^^11^^]^. Therefore, further accurate differential criteria are required for guiding the therapeutic options for these two types of malignancies. More recently, absolute quantification using flow cytometric assessment of several surface molecules have provided a potential diagnostic value in this context.

Taken together, the combination of morphologic and immunophenotypic characteristics is essential to obtain an accurate diagnosis and to minimize interpretative variations. Therefore, we aimed to first assess 131 patients with various forms of CLDs by analyzing the most useful multi-marker panel in order to achieve a clearer insight into the distinctive diagnosis of such heterogeneous disorders based on immunologic criteria.

## MATERIALS AND METHODS

**Selection of patients**

In this study, 1,185 patients were referred to Takhte Tavous Pathobiology Laboratory, Tehran, Iran. BM and peripheral blood samples were taken from the patients from April 2018 to April 2019. Among these patients, 131 (with an age ranging from 37 to 90 years) were diagnosed with lymphoproliferative diseases based on analyzing the surface markers using flow cytometry. Patients with CLDs were new cases (with no history of previous treatment) diagnosed with a chronic lymphoproliferative disease with clinical presentation of CLDs such as leukocytosis, lymphadenopathy, or splenomegaly. The exclusion criteria were patients whose chronic lympho-proliferative disease have not been diagnosed. The patients with CLDs were divided into B-CLL (n = 91), atypical B-CLL (n = 15), MCL (n = 14), and CD5-negative/CD10-negative lymphoma (n = 11) based on the expression of B-cell surface markers, including HLA-DR, CD5, CD19, CD20, CD22, CD23, CD10, CD38, CD45, and kappa and lambda light chains. Classification of CLDs was performed according to the expression of CD5, CD1, and CD23 to distinguish CLL from MCL and other chronic B-cell disorders. 

**Flow cytometry**

Fresh BM (5 ml) or blood sample was collected into vacutainer tubes containing K_2_-EDTA and gently homogenized several times. Samples were analyzed by multi-parametric flow cytometry (Beckman-Coulter FC500, USA) via MXP software using a combination of three surface markers of MoAbs in Takhte Tavous Pathobiology Laboratory. These three-color combination included (1) CD5 (FITC, Dako)/CD19 (PE, Dako)/CD23 (ECD, Coulter), (2) CD20 (FITC, Dako)/CD22 (PE, Dako)/CD38 (ECD, Coulter), (3) CD10 (FITC, Dako)/ CD19 (PE, Dako)/CD45 (PerCP-Cy5, Coulter), (4) Kappa (FITC, Dako)/Lambda (PE, Dako)/CD19 (ECD, Coulter), and HLA-DR (FITC, Dako) in a single color tube. BM or blood cells were incubated with the aforementioned MoAbs at 4 °C for 30 min, and then erythrocytes were lysed using a standard lyse/wash technique. Finally, the samples were analyzed by a flow cytometer, and antigenic expression in blast cells was systematically analyzed by multi-parametric flow cytometry. The optimal cut-off for each marker was considered as 20% expression of the analyzed events for cell surface antigen expression. 

**Immunohistochemistry**

Cyclin D1, a marker considered highly specific and sensitive for MCL was used to confirm the diagnosis of MCL. Four-micron sections of paraffin-embedded tissue blocks were stained using specific MoAb for human cyclin D1 (GeneTex, San Antonio, TX). Slides were counterstained with Harris hematoxylin and examined by standard light microscopy. Samples were analyzed by using an Olympus BX51TF microscope, and pictures were taken using Olympus QColor3 and analyzed by using QCapture 2.60 software (QImaging). We considered cyclin D1 to be positive if more than of 10% of the cells showed nuclear positivity. Cytoplasmic staining was not considered positive.

**Statistical analysis**

All statistical analyses were performed by SPSS software (version 21). We used one way ANOVA to compare the subtypes of CLDs. Correlation analysis between flow cytometric markers and hemoglobin, platelet count and leukocyte blood count were assessed by Pearson and Spearman test. P values of less than 0.05 were regarded as statistically significant.

## RESULTS

**Characteristics of patients**

In this study, 131 patients were diagnosed with B-CLL (n = 91), atypical B-CLL (n = 15), MCL (n = 14) and CD5-/CD10 ymphoma (n = 11). There were 81 (61.8%) men and 50 (38.2%) women ranging from 37 to 90 years old (mean ± SD; 65.4 ± 12.4 years). Characteristics of each group of patients, including gender, age, hemoglobin, platelet count, and leukocyte blood count, are summarized in Table 1. As shown in this Table, A significant difference in terms of hemoglobin was observed among different groups (*p* = 0.000). The patients in the CD5-/CD10-lymphoma group had lower hemoglobin content compared with the other three patient groups. Platelet count was lower in the atypical B-CLL group compared with the B-CLL, MCL, and CD5-/CD10-lymphoma groups (*p* = 0.013).

**Comparison of flow cytometry immunophenotyping markers between patient groups**

**Fig. 1:**
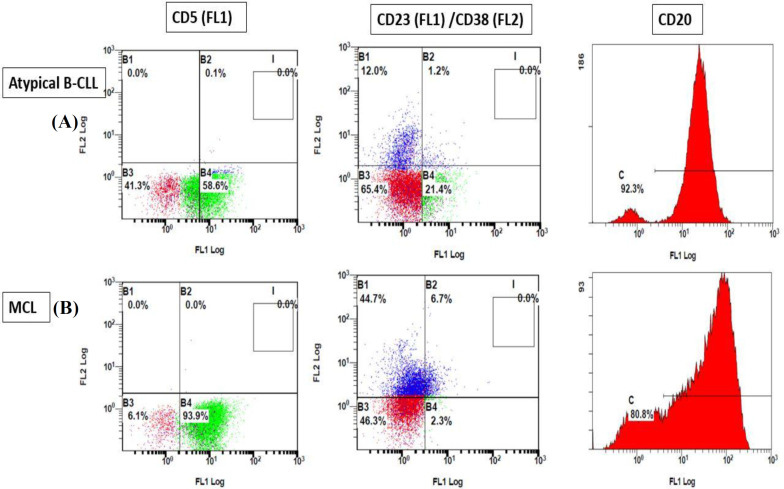
Immunophenotypic analysis in respect to the expression of CD5, CD23, CD38, and histogram of CD20 in atypical (A) B-CLL and (B) MCL. High expression of the CD5 marker was found in MCL patients, although atypical B-CLL patients expressed a lower level of this marker. Atypical B-CLL patients had CD23 expression to a less extent, but MCL patients rarely expressed this marker. Almost all patients with atypical B-CLL and MCL were negative and positive for CD38 expression, respectively. Both groups expressed CD20 without significant differences

Flow cytometric immunophenotyping studies (Table 2) showed that all groups expressed CD19 and CD20 without statistically significant changes (*p* = 0.1 and *p* = 0.2, respectively), but in the case of CD22, all the patients, except B-CLL cases, expressed this marker (*p* = 0.000). Moreover, a high expression level of the CD5 marker was found in B-CLL and MCL patients (79.89 ± 13.2 and 86.7 ± 9.6, respectively), although atypical B-CLL patients expressed a lower level of this marker (45.5 ± 27.0). Remarkably, B-CLL patients and to a less extent, atypical B-CLL patients had CD23 expression (*p* = 0.000). However, three patients with MCL expressed CD23 nearly 20%. On the other hand, only MCL, CD5-negative, and CD10-negative patients expressed FMC-7 antigen (*p* = 0.000). Interestingly, the pattern of CD22, CD23, and FMC-7 antigens, along with the CD5 co-expression, permitted the accurate classification of all B-CLL, and CD5-/CD10-lymphoma using flow cytometry. The critical challenge is that these markers are unable to differentiate MCL from atypical B-CLL; however, CD38 is another marker that can be helpful for discrimination between these two groups. In this regard, all patients with atypical B-CLL, except for one case, were negative for CD38 expression, and all MCL patients, except for one case, were positive for CD38 expression (*p* = 0.000). According to these findings, the expression of CD38 allowed us to somehow distinguish MCL from the atypical B-CLL (Fig. 1).

**Flow cytometric investigation of kappa and lambda light chain expression between MCL and atypical B-CLL**

**Fig. 2:**
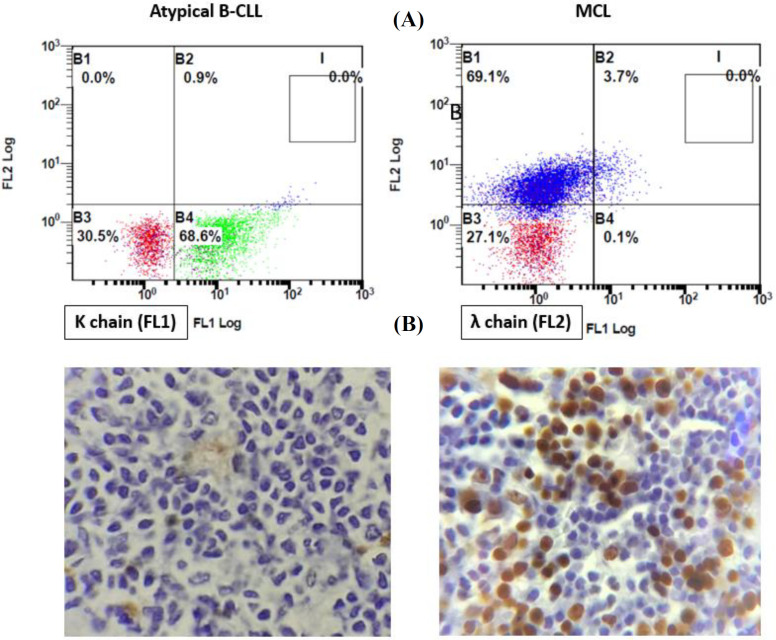
(A) Immunophenotypic analysis of light chain type in atypical B-CLL and MCL patients. Although all atypical B-CLL patients expressed kappa immunoglobulin light chain (left), most MCL patients had lambda light chain (right). (B) Micrograph of cyclin D1 staining in an atypical B-CLL patient and a patient with MCL. IHC method was conducted for cyclin D1 in atypical B-CLL patients and MCL on BM biopsy samples. All atypical B-CLL patients showed negative results for cyclin D1 (left), but it was positive in almost all MCL patients (right)

Due to the inadequacy of flow cytometric markers to precisely differentiate MCL from atypical B-CLL, we decided to evaluate the light chain type in these patients. All 15 patients with atypical B-CLL (100%) expressed kappa immunoglobulin light chain. Besides, 12 out of 14 MCL patients (85%) had lambda chain, while two cases (15%) illustrated kappa light chain. In order to measure the specificity and sensitivity of flow cytometry assessment of light chain in detecting MCL, IHC method was conducted for cyclin D1 in MCL and atypical B-CLL patients on BM biopsy samples. In cases of atypical B-CLL, all patients showed negative results for cyclin D1. Although cyclin D1 was negative in one case with lambda light chain, it was positive in the other 11 patients with lambda light expression (Fig. 2). 

**Table 1:** Characteristics of patients

**Characteristics**	**B-CLL** **(n = 91)**	**atypical B-CLL** **(n = 15)**	**MCL** **(n = 14)**	**CD5-/CD10-** **lymphoma (n = 11)**	***p*** ** value**
Gender (male/female)	58/33	8/7	8/6	7/4	0.86
Age (mean ± SD)	65.6 ± 13.7	63.9 ± 9.7	63.3 ± 7.9	68.9 ± 9.7	0.69
Hemoglobin (g/dl-mean ± SD)	12.8 ± 1.9	10.9 ± 1.9	11.6 ± 1.6	10.3 ± 1.3	0.000
Platelet count per µL (mean ± SD)	169740 ± 58528	130930 ± 57575	132290 ± 45144	140910 ± 49988	0.013
Leukocyte blood count per µL (mean ± SD)	39662 ± 40055	59400 ± 73968	29378 ± 23346	25254 ± 11769	0.156

**Table 2:** Expression of B-cell-associated antigens in patients

**Flow cytometry markers**	**B-CLL** **(n = 91)**	**Atypical B-CLL** **(n = 15)**	**MCL** **(n = 14)**	**CD5-/CD10-lymphoma (n = 11)**	***p*** **value**
CD19	76.3 ± 12.2	71.4 ± 13.4	69.3 ± 10.2	61.3 ± 15.2	0.1
CD20	70.2 ± 14.2	74.3 ± 14.1	74.3 ± 12.5	77.4 ± 11.4	0.2
CD22	18.4 ± 13.8	43.1 ± 17.9	65.2 ± 14.4	60.5 ± 10.1	0.000
CD5	79.89 ± 13.2	45.5 ± 27.0	86.7 ± 9.6	12.5 ± 5.7	0.000
CD23	66.7 ± 16.4	31.0 ± 22.8	8.2 ± 3.4	9.1 ± 6.7	0.000
FMC-7	1.3 ± 2.3	12.5 ± 9.2	40.4 ± 9.6	27.7 ± 9.8	0.000
CD38	2.5 ± 5.4	9.5 ± 8.7	65.9 ± 21.2	10.5 ± 5.3	0.000

Data are shown as mean percentage ± SD.

**Correlation between flow cytometric markers and hemoglobin content, platelet, and leukocyte** **blood counts**

**Fig. 3:**
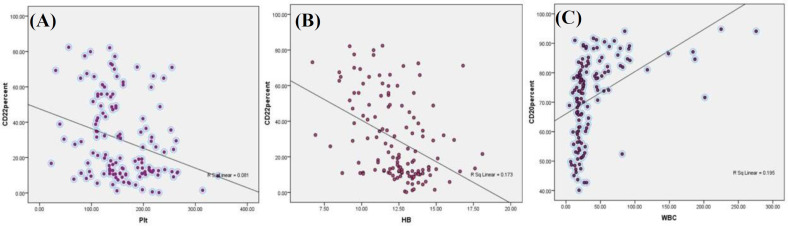
Association between (A) CD22 and platelet count, (B) CD22 and hemoglobin, and (C) CD20 and leukocyte blood count in studied patients, which all were negative

We used correlation to evaluate the association of hemoglobin, platelet count, and leukocyte blood count with flow cytometric markers, including CD5, CD20, CD22, CD23, and FMC7. As indicated in Table 3, in all experimental data, except for CD38, a strong correlation was visualized between hemoglobin and flow cytometric markers. Interestingly, correlation coefficient of CD19 and CD20 with platelet count value displayed a negative correlation with hemoglobin, platelet count, and leukocyte count (r = -0.242, *p* = 0.006 and r = -0.234, *p* = 0.007, respectively). Our results also indicated a strong negative association between CD22 (r = -0.285, *p* = 0.001), FMC-7 (r = -0.192, *p *= 0.028), and also CD38 (r = -0.176, *p* = 0.045) with platelet count. Correlation studies showed a significant positive association between CD19 (r = 0.413, *p* = 0.001) and CD20 (r = 0.42, *p* = 0.000) with leukocyte blood count (Fig. 3).

## DISCUSSION

Currently, diagnosis of B-cell CLDs is mainly based on clinical findings, morphologic characteristics, and evaluation of the immunophenotyping markers, through which the diagnosis of a large group of B-cell CLDs can be made precisely. Nevertheless, few patients still require additional investigations for differential diagnosis. In this study, we assessed a total of 131 patients with different B-cell CLDs, including B-CLL, atypical B-CLL, MCL, and CD5-/CD10-lymphoma, for a panel of B-cell-specific markers viz CD5, CD20, CD22, CD23, and FMC-7. B-CLL and MCL, both known as CD5-positive B-cell malignancies, can be differentiated mainly via CD23, which is typically absent on the surface of MCL neoplastic B-cells but is highly expressed in B-CLL. Moreover, MCL cases are often positive for FMC-7, which is commonly not expressed in B-CLL. Generally, immunophenotyping can simply differentiate B-CLL from MCL, but the differentiation of atypical B-CLL from MCL, which are immunologically very similar, remained a big challenge. This study evaluated the immunophenotype of atypical CLL and MCL, to help in the differential diagnosis of CD5-positive monoclonal B cells. D’Arena et al.^[^^12^^]^ have demonstrated the usefulness of quantitative flow cytometry to differentially diagnose the B-cell CLDs; however, no quantitative differences were found between atypical CLL and MCL. This finding further highlights the close immunophenotype of atypical B-CLL and MCL. Another study has shown that the percentage of CD54 and the median fluorescence intensity of CD20 and CD54 were significantly higher in MCL^[^^13^^]^. Moreover, recent studies have proposed that CD200 and CD43 can be valuable markers for the differentiation of atypical CLL from MCL by flow cytometry^[^^14^^-^^16^^]^. According to our findings, CD5, CD22, CD23, and FMC-7 can accurately differentiate all subgroups, except for atypical B-CLL and MCL. Therefore, we tried to provide a flow cytometric panel for the definite diagnosis of MCL, which can distinguish it from atypical B-CLL as a challenging differential diagnosis. Immunoglobulin light chains in the diagnostic cocktail can be a promising tool to discriminate between MCL and atypical-B-CLL, suggesting a challenging differential diagnosis. We demonstrated the predominant expression of lambda light chain in MCL patients (85%), a finding which is in agreement with a study performed by Bertoni et al.^[^^17^^]^ who reported lambda light chain expression in 10 out of 12 subjects with MCL (83%). However, another study represented a kappa restriction in indolent MCL patients^[^^18^^]^. Overall, according to the guidelines for investigation of MCL, the classical MCL is lambda light chain-restricted, while the indolent MCL is mostly kappa-restricted^[^^19^^]^.

**Table 3:** Correlation between flow cytometric markers and hemoglobin level, platelet count, and leukocytes blood count

**Flow cytometric** **markers**	**Hemoglobin** **r (** ***p*** ** value)**	**Platelet count** **r (** ***p*** ** value)**	**Leukocyte blood count ** **r (** ***p*** ** value)**
CD19	-0.261 (0.001)	-0.242 (0.006)	0.413 (0.001)
CD20	-0.275 (0.002)	-0.234 (0.007)	0.442 (0.000)
CD22	-0.416 (0.000)	-0.285 (0.001)	0.090 (0.30)
CD5	0.284 (0.001)	0.102 (0.24)	0.124 (0.15)
CD23	0.302 (0.000)	0.144 (0.10)	0.142 (0.10)
FMC-7	-0.245 (0.005)	-0.192 (0.28)	-0.033 (0.71)
CD38	-0.141 (0.10)	-0.176 (0.045)	-0.081 (0.36)

Our results indicated that exploring the pattern of kappa and lambda immunoglobulin light chain expression by flow cytometry can distinguish MCL from atypical B-CLL with a high sensitivity (91%) and moderate specificity (50%); as all cases (100%) of atypical B-CLL expressed kappa light chain, and most cases (85%) with MCL had lambda light chain expression. The sensitivity and specificity were calculated in comparison with cyclin D1 expression using IHC method, as a specific and sensitive method for MCL diagnosis. We also assessed the correlation of hemoglobin, platelet, and leukocyte blood count with the immunophenotyping markers. Our results showed that CD20 had a negative correlation with hemoglobin level and platelet count, while it was positively correlated with leukocyte blood count. As a result, the higher CD20 expression is associated with the lower hemoglobin content and platelet number and also with the higher leukocyte blood count. In a recent study, Asnafi et al.^[^^20^^]^ displayed a significant negative correlation between CD20 expression and platelet counts. Furthermore, we found that the high expression of CD22, similar to CD20, was related to the low levelof both hemoglobin and platelet count. Our data also revealed a negative correlation between FMC-7 and hemoglobin, while CD5 and CD23 were positively correlated with hemoglobin content. Generally, our findings highlight the clinical importance of the quantitative expression of the immunophenotyping markers and their probable value as diagnostic markers in different subgroups of B-cell CLD patients. Altogether, expression of CD20 (bright), CD22, FMC-7, CD38, and lambda light chain, along with positivity in CD5 expression and negativity in expression of CD23, can confirm MCL detection and differentiate it from atypical B-CLL.

## DECLARATIONS

### Acknowledgments

We acknowledge the staff of Takhte Tavous Pathobiology Lab, Tehran, Iran for data collection and technical support.

### Ethical statement

The above-mentioned sampling protocol was approved by the Medical Ethics Committee of Shahid Beheshti University of Medical Sciences, Tehran, Iran (IR.SBMU.REC.1398.95). A written consent was obtained from each patient. 

### Data availability

The analyzed data sets generated during the study are available from the corresponding author on reasonable request.

### Author contributions

MM: evaluated the data and drafted the manuscript; MSHJ: re-evaluated the data and revised manuscript; LJ: revised manuscript; MM: evaluated the data; ESS: designed the study and collected the data. 

### Conflict of interest

None declared.

### Funding/support

This study has received no financial support from any institute or organization. 
